# Bilateral Supplemental Permanent Maxillary Lateral Incisor in a Non-syndromic Pediatric Patient: A Rare Case Report

**DOI:** 10.7759/cureus.67254

**Published:** 2024-08-19

**Authors:** Rutuja Patil, Harikishan Kanani, Monika Khubchandani, Ramakrishna Yeluri

**Affiliations:** 1 Pedodontics and Preventive Dentistry, Datta Meghe Institute of Higher Education and Research, Sharad Pawar Dental College, Wardha, IND; 2 Pediatric Dentistry, Sharad Pawar Dental College and Hospital, Datta Meghe Institute of Higher Education and Research, Wardha, IND; 3 Pediatric Dentistry, Sharad Pawar Dental College, Datta Meghe Institute of Medical Sciences, Wardha, IND

**Keywords:** pediatric dentistry, pediatric patient, supplemental lateral incisors, non syndromic, supernumerary lateral incisor, supernumerary teeth

## Abstract

In the present case report, a 13-year-old girl patient has bilateral supplementary maxillary lateral incisors, a rare type of supernumerary teeth. During a regular checkup, it was discovered that the patient had these additional teeth, although she had no notable medical or dental history. The patient had no symptoms, even though they may have led to occlusal problems. The tooth's morphology was identical to that of the nearby natural teeth, and radiographic imaging verified full root growth. The example illustrates the importance of early diagnosis and individualized treatment planning, taking into account the patient's decision to keep the extra teeth while attending to the main issue of widespread dental sensitivity. This study emphasizes the necessity for tailored patient treatment and the variations in the management of extra teeth.

## Introduction

One or more teeth on one or both sides of the dental arches may be supernumerary; the premaxilla is the most typical location [1]. The exact etiology of hyperdontia remains unknown [2]. Numerous theories explain why it occurs, including the dichotomy theory, the atavism hypothesis, and the dental lamina hyperactivity theory [2]. The most well-known explanation is the hyperactivity theory, which contends that the creation of supernumeraries is mostly based on the local, independent, conditioned heightened activity of the dental lamina [3].

The frequency of extra teeth in the deciduous dentition is 0.3%-0.6%, five times less than in the permanent dentition. Deciduous teeth are thought to occur less frequently as a result of their extraction or exfoliation [4]. Compared to the lower jaw, the maxilla forms eight times as many supernumerary teeth [5]. Among the Supernumeraries, permanent lateral supernumerary incisors are not prevalent [6].

According to research, the frequency of supernumerary teeth in secondary dentition ranges from 0.5% to 5.3% [7]. The majority of afflicted men have more than one extra tooth compared to women, with a documented 2:1 ratio. However, when it comes to primary dentition, there are no appreciable differences in the gender distribution [8].

Conical, tuberculate, supplementary, and odontoma are the four morphological categories. Supplemental is a form of supernumerary teeth which is located toward the end of the teeth series and is shaped and sized very similarly to a regular tooth. The maxillary permanent lateral incisor is the most commonly occurring supplemental tooth. Moreover, this type of tooth makes up the majority of the extra teeth recorded in the primary dentition [9]. In this case, the bilateral supplemental lateral incisors are seen with the upper arch.

Supplemental lateral incisors may be erupted or not in the permanent or primary dentition and they may be bilateral or unilateral [10-12]. The aesthetics of the front dentition may be negatively impacted by supplemental lateral incisors, which can also result in occlusal abnormalities such as severe overjet, crowding or ectopic eruption, midline shift, and low self-esteem [2].

In any case, if the additional teeth erupt or remain impacted, there may be clinical problems. In general, these teeth may be the source of the following problems: over-retained primary teeth, permanent teeth that have impacted or delayed eruption, tooth rotations or displacements, pathological characteristics such as the formation of odontogenic cysts (dentigerous cysts, follicular cysts), abnormal diastema, or the rare resorption of the roots are all examples of dental abnormalities. The most important and frequent outcome that might occur if the patient's teeth are left untreated is root resorption of the nearby permanent teeth [13].

## Case presentation

**Figure 1:**
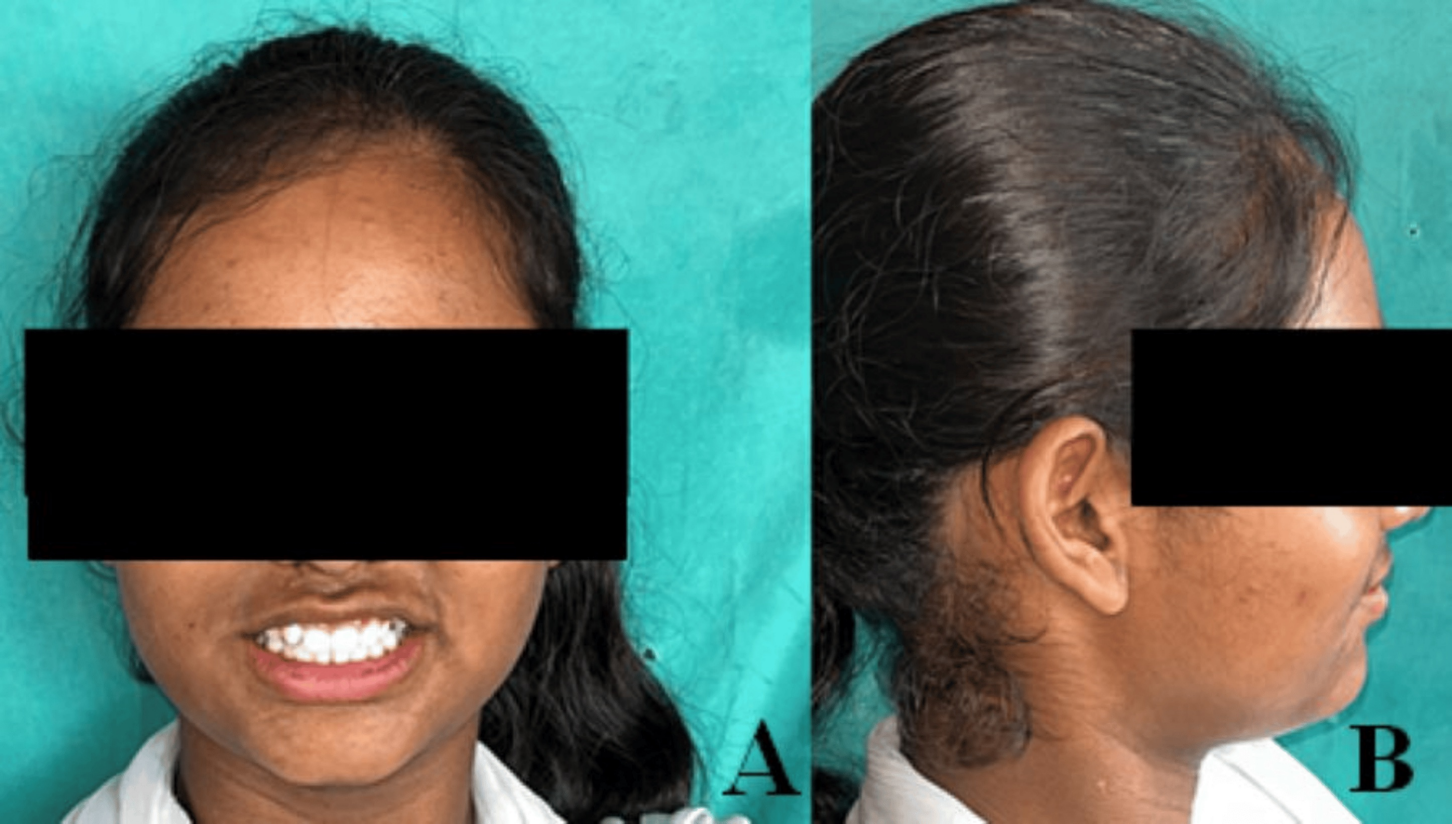
Extraoral photographs. (A) Front view. (B) Lateral view.

A 13-year-old female patient reported to the Department of Pediatric and Preventive Dentistry with a chief complaint of generalized tooth sensitivity (Figures 1A, 1B). The patient had no contributing medical history; there was no abnormality found during the extra-oral examination, and there was no prior history of any injury to the teeth or jaws. The intraoral examination of the permanent dentition reveals no abnormalities related to the primary treatment.

**Figure 2:**
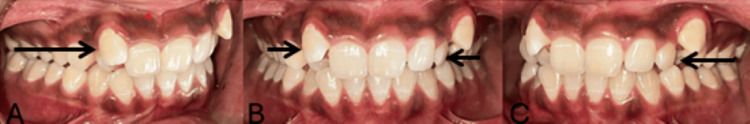
Intraoral occlusal view photographs. (A) Right side. (B) Front side. (C) Left side.

**Figure 3:**
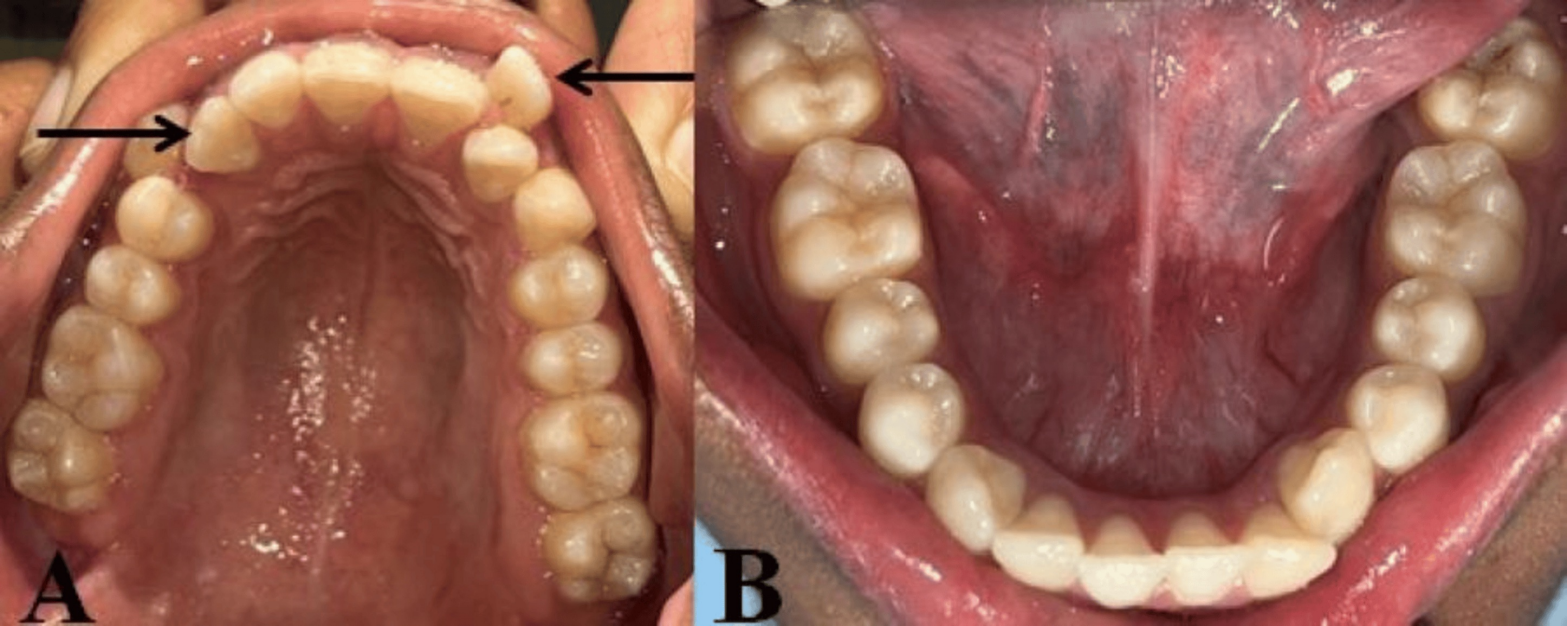
Intraoral photograph. (A) Maxillary arch. (B) Mandibular arch.

However, coincidentally, an extra incisor with morphology comparable to the lateral incisor was observed on the right and left sides of the upper arch, between 11 and 12 and 21 and 22, respectively. There was no interference with occlusion in this instance; the patient had Class I molar occlusion. With upper anterior crowding, the upper permanent right canine is present in the arch, and the left canine erupted buccally in the arch (Figures 2A-2C, 3A, 3B).

**Figure 4:**
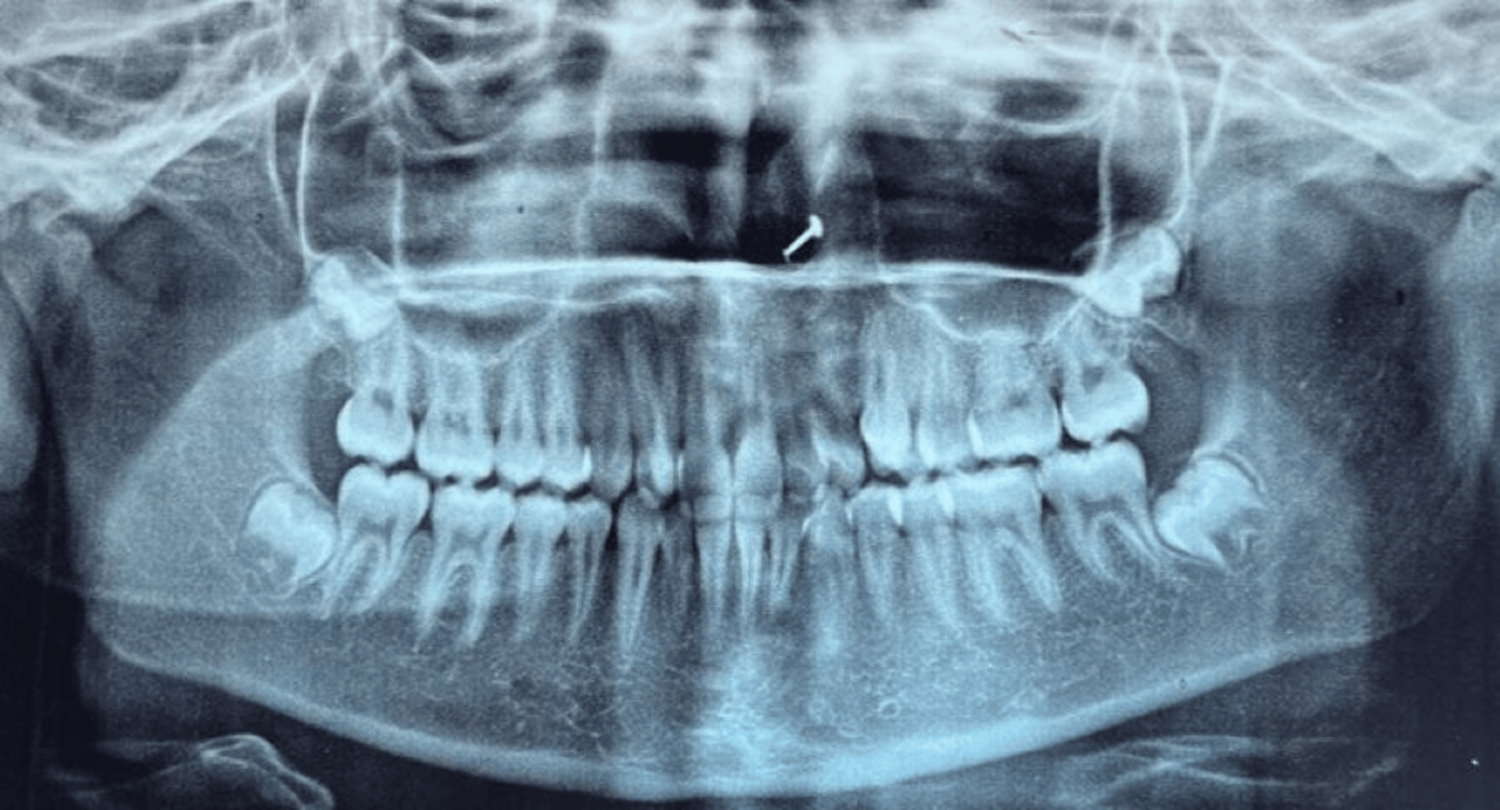
Orthopantomogram showing complete root development.

In this circumstance, the patient had no complaints. Upon evaluation with an orthopantomography, complete root development was seen with upper right and left supplementary teeth. The supplementary teeth appeared to be typical in size and shape, and they were situated between 11 and 12 and 21 and 22 (Figure 4).

In this case, the diagnosis was hyperdontia, and the options for treatment included extraction of the supplementary permanent maxillary left and right lateral incisors and fixed orthodontic alignment of the upper teeth. Despite being informed of the situation and the proposed course of treatment, the patient decided to retain the extra teeth. As a result, the additional teeth were left in place. The patient's main complaint was teeth sensitivity. To address that, desensitizing toothpaste and mouthwash were prescribed for 15 days.

## Discussion

Both the primary and permanent dentition may have supernumerary teeth. Additionally, syndromic patients may have an additional tooth, and non-syndromic people may have sporadic instances of this condition. Around 12%-23% of non-syndromic instances have more than one supernumerary tooth, whereas 76%-86% of cases involve just one [14]. Because they resemble the neighboring natural teeth and do not seem to be causing any problems, the patient in this case report was unaware that there were additional permanent maxillary lateral incisors. Studies show that the development of extra lateral incisors is an uncommon occurrence, with bilateral examples being much more unusual [15].

The dental laminate hyperactivity theory is the most widely recognized explanation for the etiology of extra teeth, despite several attempts to do so. Supernumerary teeth may also result from inheritance patterns and genetic causes [3]. The course of therapy in these situations depends on the underlying malocclusion. Identification of extra teeth by radiography and clinical means is essential for treatment planning [3].

According to research, the supplementary teeth are distributed based on their positions. The results showed that there were 24 (21.4%) teeth in the arch, 30 (26.7%) teeth that were labially/buccally displaced to the arch, 30 (32.1%) teeth that were palatally/lingually displaced, seven (6.2%) teeth that were positioned at the distal end, and 15 (13.3%) teeth that were impacted [13]. A supplemental tooth may exhibit a deep palatal pit and coronal invagination in comparison to the original tooth. In this case, the right supplemental lateral incisor is present buccally, while the left one is positioned in the arch [13].

According to Barbara et al., 18 individuals were found to have 20 supernumerary lateral incisors. According to their analysis, 10 patients had a midline shift from having too many teeth; four had a severe space deficit; one had an ectopic eruption; and four had an extreme overjet. The permanent supernumerary incisors had not yet erupted in three individuals, while the tooth was still retained in one patient [16].

Developmental abnormalities may include the incidence of extra teeth. Cleft lip and palate are the most prevalent disorders that have a notable prevalence of numerous supernumerary teeth [17]. Other conditions include chondroectodermal dysplasia, Gardner's syndrome, and cleidocranial dysostosis [18]. However, none of the previously listed disorders were present in the patient who was seen here. This instance showed a supernumerary tooth was unrelated to any condition. Any possible issues that may arise throughout the child's growth can be found by a radiographic evaluation of both dental arches [18]. If a supernumerary tooth that is inhibiting permanent tooth eruption is removed, the tooth will often emerge if there is enough room [6].

Treatment of supernumerary teeth depends on whether or not they have erupted. In cases when the tooth is fully erupted, extraction of the supplemental lateral incisors is recommended, if the tooth has any pathologic, cosmetic, or occlusal problems. When a tooth erupts normally and does not create any occlusal or aesthetic disruption to the arch, extraction is not the recommended course of action. In certain cases, it is preferable to keep the additional tooth in place while keeping an eye on it. If these teeth are not erupting, it is possible to look into whether or not they are linked to any problems. Sometimes it is advisable to keep them under monitoring but leave them in place if there are no difficulties [19]. On the other hand, the unerupted supernumerary tooth has to be surgically removed if it is linked to any complications like pain and swelling, or pathological problems like odontogenic cysts.

## Conclusions

This case highlights the importance of thorough clinical and radiographic evaluations in identifying incidental findings, such as supernumerary teeth. The presence of bilateral supplemental maxillary lateral incisors in a non-syndromic patient is rare and can be easily overlooked due to their resemblance to natural teeth. Treatment decisions should be individualized, taking into consideration the patient's preferences and potential functional and aesthetic impacts. In this case, the patient's decision to retain the extra teeth emphasizes the necessity of patient-centered care in dental practice.
